# Novel function of the poly(c)-binding protein α-CP2 as a transcriptional activator that binds to single-stranded DNA sequences

**DOI:** 10.3892/ijmm.2013.1488

**Published:** 2013-09-11

**Authors:** DUK-HEE KANG, KYU YOUNG SONG, LI-NA WEI, PING-YEE LAW, HORACE H. LOH, HACK SUN CHOI

**Affiliations:** 1 Division of Nephrology, Department of Internal Medicine, Ewha Medical Research Institute, Ewha Womans University School of Medicine, Yangcheon-gu, Seoul 158-710, Republic of Korea; 2 Department of Pharmacology, University of Minnesota Medical School, Minneapolis, MN 55455, USA

**Keywords:** α-complex protein 2, post-transcriptional regulation, transcriptional regulation, electrophoretic mobility shift assays, poly(C) sequence, transcriptional activator

## Abstract

α-complex protein 2 (α-CP2) is known as an RNA-binding protein that interacts in a sequence-specific manner with single-stranded polycytosine [poly(C)]. This protein is involved in various post-transcriptional regulations, such as mRNA stabilization and translational regulation. In this study, the full-length mouse α-CP2 gene was expressed in an insoluble form with an N-terminal histidine tag in *Escherichia coli* and purified for homogeneity using affinity column chromatography. Its identity was confirmed using matrix-assisted laser desorption/ionization time-of-flight (MALDI-TOF) mass spectrometry. Recombinant α-CP2 was expressed and refolded. The protein folding conditions for denatured α-CP2 were optimized. DNA and RNA electrophoretic mobility shift assays demonstrated that the recombinant α-CP2 is capable of binding to both single-stranded DNA and RNA poly(C) sequences. Furthermore, plasmids expressing α-CP2 activated the expression of a luciferase reporter when co-transfected with a single-stranded (pGL-SS) construct containing a poly(C) sequence. To our knowledge, this study demonstrates for the first time that α-CP2 functions as a transcriptional activator by binding to a single-stranded poly(C) sequence.

## Introduction

The polycytosine [poly(C)]-binding proteins (PCBPs) are characterized by heterogeneous nuclear ribonucleoprotein (hnRNP) K homology (KH) domains and high affinity sequence-specific interactions with polycytosine [poly(C)] nucleic acid sequences. In mammalian cells, five evolutionarily related PCBPs have been identified: PCBP1–4 and hnRNP K (1). These PCBPs belong to one of two subgroups: hnRNP K or the α-complex proteins (α-CPs or PCBP1–4) (2). Each PCBP has three KH domains. hnRNP K, PCBP1 (α-CP1 or hnRNPE1), and PCBP2 (α-CP2 or hnRNPE2) have been extensively investigated (3,4). Two other members of the α-CP family have also been discovered: PCBP3 (α-CP3) and PCBP4 (α-CP4) (5).

All members of the PCBP family are related evolutionarily. The common feature of all PCBPs is the presence of three hnRNP KH domains (6). These are RNA-binding modules that are approximately 70 amino acids in length. The KH domain of PCBPs consists of three α-helices and β-strands arranged as follows: β1-α1-α2-β2-β3-α3 (7). The Gly-X-X-Gly loop is located between α1 and α2, and the variable loop is located between β2 and β3. These KH domain sequences are conserved in PCBPs (7,8). PCBP1 and PCBP2 share the highest level of amino acid sequence similarity (89%). PCBP3 is more divergent, and PCBP4 is the most distantly related (52% divergence from α-CP2) (5,9).

The function of PCBPs is dependent on their localization to either the cytoplasm (mRNA stability and translational regulation) or nucleus (transcription and splicing). PCBP1 and PCBP2 are primarily localized in the nucleus and nuclear speckles. By contrast, PCBP3 and PCBP4 are primarily localized in the cytoplasm (9). The signal-dependent post-translational modifications of PCBPs can regulate their ability to bind nucleic acids. For example, the phosphorylation of PCBP1 and PCBP2 markedly decreases their RNA-binding activity (3), and the phosphorylation of PCBP1 increases its DNA-binding activity (10). Another important determinant of the different functions of PCBPs is their subcellular localization (9,10). PCBP1 has been shown to function as a cytosolic iron chaperone during the delivery of iron to ferritin. Such iron binding to PCBP1 may significantly alter its nucleic acid binding activity (11). PCBP2 can participate in protein-protein interactions (12) and has been linked to the regulation of poliovirus replication (13); it also plays a role in innate immunity (14). PCBP4 (MCG10) can induce apoptosis (15) and may function as a lung tumor suppressor and its expression can inhibit the proliferation and tumorigenesis of lung cancer cells, both *in vivo* and *in vitro*, by delaying the progression of the cell cycle (16,17). Members of this family perform multiple functions by binding to poly(C) sequences, including mRNA stabilization (18–20), translational silencing (21,22) and translational enhancement (19,23).

In this study, we report the purification, refolding and characterization of an α-CP2 protein that binds to single-stranded DNA and RNA poly(C) sequences. We purified recombinant α-CP2 using affinity column chromatography and confirmed its identity using mass spectrometry. This study demonstrates a dual binding function for the α-CP2 protein via specific interactions with single-stranded DNA and RNA poly(C) sequences. To our knowledge, we also demonstrate for the first time that α-CP2 functions as a transcriptional activator by binding to single-stranded poly(C) sequences.

## Materials and methods

### Plasmid construction

The single-strand forming construct, pGL-SS, was generated by ligating an annealed double-stranded oligonucleotide into the *Sac*I and *Hin*dIII sites of pGL3-basic (Promega, Madison, WI, USA) using the following oligonucleotide sequences: 5′-ATTGAGCTCACAATCCACTCCTTCTCTCTCCTCCCTCCCCTCTAGCCTCTGAAGCTTTTC-3′) (sense) containing a *Sac*I site (underlined) and 5′-GAAAAGCTTCAGAGGCTAGAGGGGAGGGAGGAGAGAGAAGGAGTGGATTGTGAGCTCAAT-3′ (antisense) containing a *Hin*dIII site (underlined). To clone the α-CP2 gene, total RNA was isolated from mouse NS20Y cells. RNA was treated with RNase-free DNase (Promega) according to the manufacturer's instructions. RT-PCR was performed using the OneStep RT-PCR kit (Qiagen, Valencia, CA, USA). PCR was performed with primers that were designed using the gene sequence information for each protein: α-CP2 (Gene ID 6997238) 5′-AACTGCTAGACATGGACACCG-3′ (sense) and 5′-AGGTGGCATGGGTAGCAGCTAG-3′ (antisense). The PCR conditions were as follows: 94°C for 3 min; 35 cycles of 94°C for 1 min, 55°C for 1 min, and 72°C for 1 min; and 72°C for 10 min. The RT-PCR products were excised from a 1% agarose gel, purified using a QIAquick gel extraction kit (Qiagen), and cloned into a pCRII-TOPO vector (Invitrogen, Carlsbad, CA, USA). The candidate plasmids containing inserts of the correct size were confirmed using restriction enzyme digestion and DNA sequencing on an ABI 3100 sequencer (Applied Biosystems, Cambridge, MA, USA). For the transient expression studies, the α-CP2 gene was cloned by digesting the above pCRII-TOPO PCBP2 clone with 5′-*Hin*dIII and 3′-*Xho*I into the same sites of a pcDNA4 vector (Invitrogen), generating a pcDNA4-α-CP2 plasmid. The DNA sequences of all constructs were confirmed by DNA sequencing. For the protein expression experiments with *Escherichia coli* (*E. coli)*, the α-CP2 gene was cloned by digesting the above pcDNA4-α-CP2 plasmid with 5′-*Hin*dIII and 3′-*Xho*I into the same sites of a pET21b vector, generating a pET21b-α-CP2 plasmid. The DNA sequences of all constructs were confirmed by DNA sequencing.

### α-CP2 protein expression

Protein was expressed in LB medium containing ampicillin (50 μg/ml). To obtain the protein, several cell growth conditions were generated by varying the temperature and isopropylthio-β-galactoside (IPTG) concentration. Typically, 2 ml of an overnight culture were added to 100 ml of medium and incubated with vigorous shaking at a temperature in the range of 37°C. When the culture reached OD_600_=0.5, protein expression was induced with 1 mM IPTG. The samples were further incubated for 4 h after induction. The cells were harvested by centrifugation at 4,000 × g for 10 min at 4°C, washed with TE buffer (10 mM Tris-HCl, 1 mM EDTA, pH 8.0) and stored at −80°C.

### Preparation of inclusion bodies and purification of recombinant α-CP2 protein

The cell pellet was resuspended in 30 ml of buffer A (20 mM Tris-HCl, 100 mM NaCl, 1 mM PMSF, pH 7.0, containing 10 μl of 1 mg/ml DNase I) and sonicated at 4°C with 5 cycles. The lysate was centrifuged at 10,000 × g for 15 min at 4°C. The pellet was resuspended in 5 volumes of buffer A, stirred at room temperature for 5 min and centrifuged at 10,000 × g for 15 min at 4°C. The inclusion bodies were then washed three times with 10 volumes of 20 mM Tris-HCl containing 100 mM NaCl at pH 7.0. The inclusion body pellet was resuspended in 30 ml of buffer B (50 mM NaH_2_PO_4_, 300 mM NaCl, pH 8.0, 8 M urea) to solubilize the inclusion bodies. Sonication was necessary to suspend the pellet. The suspension was then centrifuged at 10,000 × g for 20 min, and the supernatant was transferred to a clean tube. The supernatant was then added to an equilibrated Ni-NTA column and allowed to drain via gravity flow. The column was washed with buffer B, and the His-tagged α-CP2 was eluted using an elution buffer (50 mM NaH_2_PO_4_, 300 mM NaCl, 250 mM imidazole, pH 8.0, 8 M urea). To determine which fractions contain the His-tagged α-CP2, we analyzed an aliquot of each sample using 10% SDS-PAGE.

### Folding of the α-CP2 protein

The washed inclusion bodies were resuspended in 5 volumes of buffer C (20 mM Tris-HCl, 1 mM EDTA, 10 mM DTT, 8 M Urea, pH 7.0), stirred at room temperature for 60 min and centrifuged at 10,000 × g for 15 min at room temperature. The pellet was discarded and the supernatant (5–10 mg/ml) was collected in a new tube. The refolding experiments were performed using protein-folding spin-columns following the manufacturer's recommendations (ProFoldin, Hudson, MA, USA).

### SDS-PAGE, in-gel tryptic digestion and matrix-assisted laser desorption/ionization time-of-flight (MALDI-TOF) mass spectrometric analysis of α-CP2

The purified α-CP2 protein was resolved on a 10% SDS-PAGE gel. The Coomassie blue-stained gel was destained, and a gel slice containing the band of interest was subjected to in-gel tryptic digestion as previously described (24). The tryptic peptides were extracted with 5% acetic acid followed by 5% acetic acid and 50% acetonitrile. The samples were dissolved in 5% acetic acid and desalted using ZipTip™ C18 reverse-phase desalting Eppendorf tips (Millipore, Billerica, CA, USA). The peptides were eluted with 2% acetonitrile containing 0.1% trifluoroacetic acid (TFA) in a volume of 20 μl. The samples were analyzed using a MALDI-TOF mass spectrometer (Applied Biosystems). The masses of the monoisotopic peaks were compared to a theoretical digestion of the protein by trypsin. The Mascot database searching software (Matrix Science, http://www.matrixscience.com) was used to identify the binding proteins.

### RNA electrophoretic mobility shift assay (EMSA)

EMSA was performed as previously described (25). The single-stranded RNA probe (5′-CUCUCCUCCCUCCCCUCUAGCCUC-3′) was end-labeled with [γ-^32^P] dATP. The free nucleotides were separated by centrifugation through a Sephadex G-25 column (Roche Diagnostics, Indianapolis, IN, USA). The end-labeled single-stranded RNA probe was incubated with recombinant α-CP2 (0.5 μg) in a final volume of 20 μl of RNA EMSA buffer [10 mM Tris (pH 7.8), 10% glycerol, 0.5 mM EDTA, 1 mM MgCl_2_, 0.1 mg/ml bovine serum albumin, 0.5 mg/ml yeast tRNA and 5 units of RNAsin] at room temperature for 20 min. For the oligonucleotide competition analyses, a 100-fold molar excess of a cold competitor RNA oligonucleotide was added to the mixture prior to adding the probe. The reactions were then incubated at 4°C for 30 min. The reaction mixtures were electrophoresed on a non-denaturing 4% polyacrylamide gel in 0.5X TBE (45 mM Tris-borate and 1 mM EDTA) at 4°C and visualized by autoradiography.

### DNA EMSA

EMSA was performed as described in a previous study (26). The single-stranded probe (5′-CAATCCACTCCTTCTCTCTCCTCCCTCCCCTCTAGCCTCTG-3′) was end-labeled with [γ-^32^P] dATP. The free nucleotides were separated by centrifugation through a Sephadex G-25 column (Roche Diagnostics). The end-labeled single-stranded DNA probes were incubated with recombinant α-CP2 (0.5 μg) in a final volume of 20 μl of EMSA buffer [10 mM Tris (pH 7.5), 5% glycerol, 1 mM EDTA, 50 mM NaCl, 1 mM DTT, 0.1 mg/ml poly(dI-dC)] at room temperature for 20 min. For the oligonucleotide competition analyses, a 100-fold molar excess of a cold competitor oligonucleotide was added to the mixture prior to adding the probe. The reactions were then incubated at 4°C for 30 min. The reaction mixtures were electrophoresed on a non-denaturing 4% polyacrylamide gel in 0.5X TBE at 4°C and visualized by autoradiography.

### S1 nuclease sensitivity assay

The pGL-SS plasmid was digested with various amounts of S1 nuclease (Promega) in S1 nuclease buffer for 15 min at 37°C. The digestion was terminated by phenol/chloroform extraction and the plasmids were recovered by precipitation. The resulting S1-treated plasmids were then digested further using *Xba*I, and the products were resolved by electrophoresis on a 1% agarose gel.

### Transient transfection and reporter gene assays

Mouse NS20Y neuroblastoma cells were grown in Dulbecco's minimum essential medium supplemented with 10% heat-inactivated fetal bovine serum at 37°C in a humidified atmosphere of 5% CO_2_. The NS20Y cells were plated in 6-well dishes at a concentration of 0.5×10^6^ cells/well and cultured overnight prior to transfection. Equimolar concentrations of various plasmids were transfected using the Effectene transfection reagent (Qiagen) as previously described (27). Briefly, for the luciferase analysis of the pGL-SS promoter, 0.5 μg of the reporter plasmids was mixed with the Effectene transfection reagent for 10 min before being added to the NS20Y cells. Forty-eight hours after transfection, the cells grown to confluence were washed once with phosphate-buffered saline and lysed with lysis buffer (Promega). To correct for differences in transfection efficiency, a one-fifth molar ratio of pCH110 (Amersham Biosciences, Piscataway, NJ, USA) containing the β-galactosidase gene under the SV40 promoter was included in each transfection for normalization. The luciferase and β-galactosidase activities of each lysate were determined according to the manufacturer's recommendations (Promega and Tropics, respectively).

### Western blot analysis

The proteins isolated from the NS20Y cells transfected with the α-CP2 gene were incubated with treatment buffer [62.5 mM Tris-HCl (pH 6.8), 2% SDS, 10% glycerol, 5% 2-mercaptoethanol] and boiled for 5 min. The treated extracts were resolved by SDS-PAGE using a 12% polyacrylamide gel. The gels were electroblotted onto polyvinylidene difluoride membranes (Amersham Biosciences) in a transfer buffer (48 mM Tris-HCl, 39 mM glycine, 20% methanol). The membranes were blocked in a blocking solution (10% dry milk and 0.1% Tween-20 in Tris-buffered saline) overnight at 4°C. Western blot analysis with anti-Myc (Santa Cruz Biotechnology, Inc., Santa Cruz, CA, USA) and anti-β-actin antibodies (Cell Signaling Technology, Beverly, MA, USA) was performed according to the manufacturer's instructions (Amersham Biosciences). The signals were detected using a Storm 840 PhosphorImager system (Amersham Biosciences).

## Results

### Expression and purification of α-CP2

**Figure 1:**
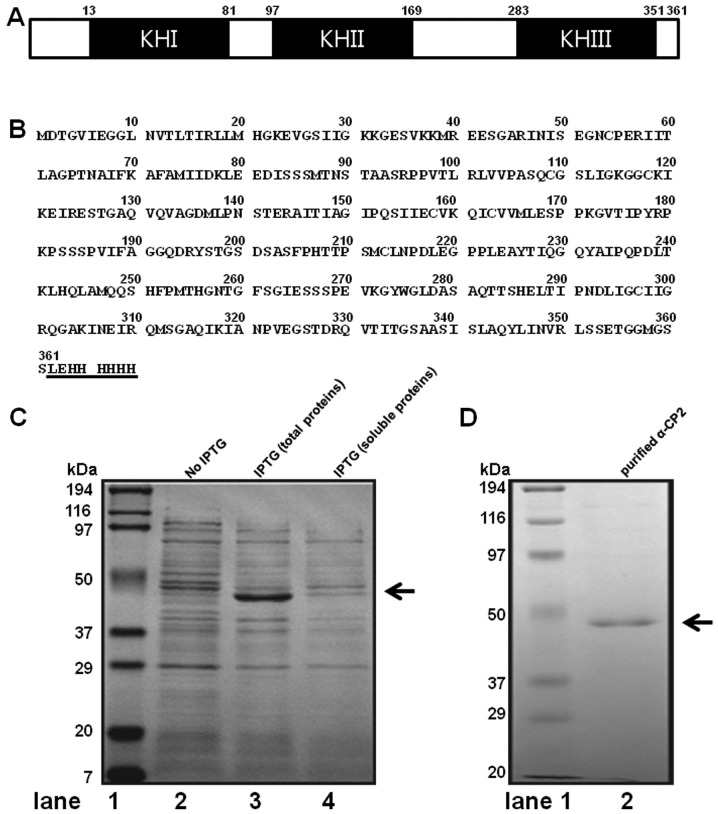

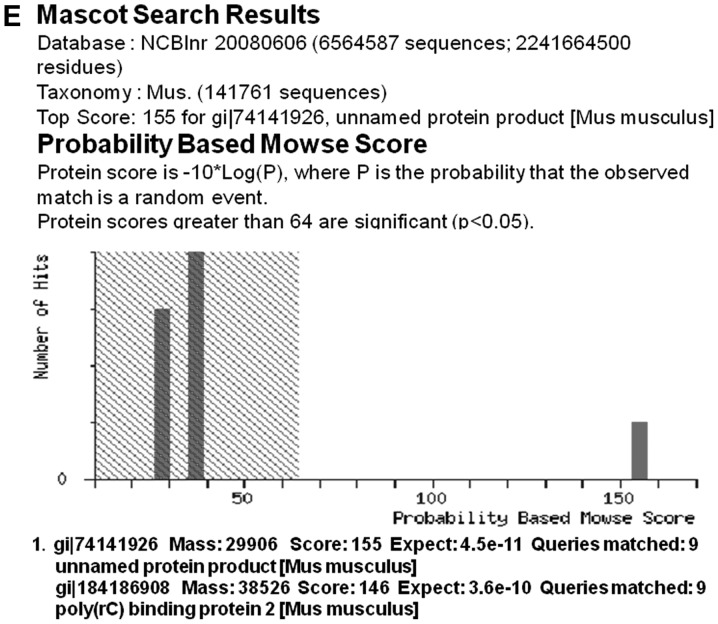
Expression and purification of recombinant α-complex protein 2 (α-CP2). (A) A diagram of the domain structure of α-CP2. hnRNPK homology (KH) domains I–III (shaded boxes). (B) The peptide sequence of α-CP2 with the His-tag fused at the carboxyl terminus. (C) SDS-PAGE analysis of recombinant mouse α-CP2 protein expressed in *E. coli*. Induced expression system (1 mM IPTG at 37°C). Lane 1, protein molecular weight markers; lane 2, 5 μl of total protein from *E. coli* BL21(DE3)/pET21b-α-CP2 before induction; lane 3, 5 μl of total protein from *E. coli* BL21(DE3)/pET21b-α-CP2 after induction; lane 4, 5 μl of soluble protein from *E. coli* BL21(DE3)/pET21b-α-CP2 after induction. (D) SDS-PAGE analysis of recombinant purified α-CP2 protein expressed in *E. coli*. Lane 1, protein molecular weight markers; lane 2, 5 μl of affinity-purified recombinant mouse α-CP2 protein after induction (arrow). (E) Mascot results from the mass spectrometric analysis of the purified protein. The value with the highest score (score of 155) identifies the protein as mouse α-CP2.

α-CP2 contains three hnRNPK homology (KH) domains, two consecutive KH domains at the amino terminus and a third KH domain at the carboxyl terminus, separated by an intervening sequence. The mouse α-CP2 gene encodes a 361 amino acid protein with a calculated molecular mass of 38,150 Da and a pI of 6.61 (Fig. 1A and B). The mouse α-CP2 gene was cloned into the pET21b vector, resulting in the expression of a recombinant α-CP2 with a 6xHis-tag at the C-terminus (α-CP2-His). The conditions for expressing the soluble protein of α-CP2 in the *E. coli* strain BL21(DE3) were extensively tested, including the temperatures for cell growth, cell culture mediums (LB and 2xYT), and induction at different stages of growth. However, almost all the conditions produced the inclusion body of the α-CP2 protein. To obtain the maximum amount of insoluble α-CP2-His protein, the expression conditions were optimized by a series of trials. The highest percentage of insoluble protein was obtained when the expression of the α-CP2-His protein was induced by 1 mM IPTG at 37°C for 4 h. Under the optimal condition, approximately 30% of the α-CP2-His was present in the insoluble fraction, as analyzed by SDS-PAGE with Coomassie brilliant blue staining (Fig. 1C). His-tags are excellent tools for purifying recombinant proteins from crude *E. coli* extracts, and immobilized metal affinity chromatography is the most commonly used method for purifying recombinant proteins containing a short 6xHis-tag. Thus, Ni-NTA His-binding resin affinity chromatography was employed to purify the insoluble recombinant α-CP2 under 8 M Urea. After washing with washing buffer, the protein was eluted with 250 mM imidazole. The SDS-PAGE results (Fig. 1D, lane 2) revealed that the α-CP2-His protein was a single band. The molecular weight was estimated to be 45 kDa. To confirm that we had isolated the α-CP2 protein, we analyzed the purified band using MALDI-TOF mass spectrometry and bioinformatics. Based on its high score (score of 155) on the Mascot search results (Fig. 1E), the protein was identified as mouse α-CP2.

### Folding of α-CP2

**Figure 2:**
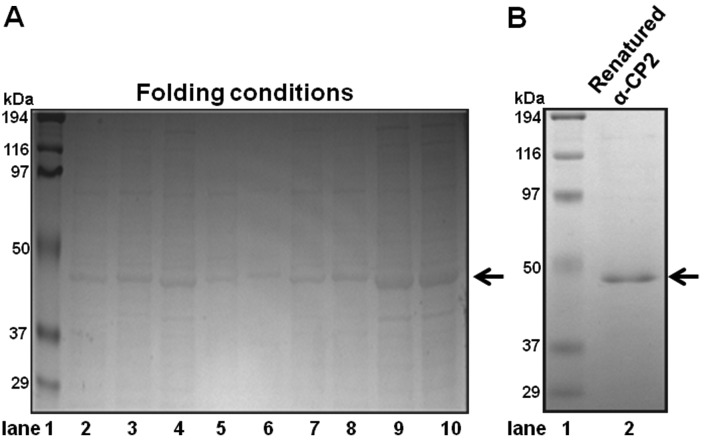
Folding of the denatured α-complex protein 2 (α-CP2). (A) Optimization of folding conditions for the purified recombinant mouse α-CP2. The solubilized inclusion bodies (5–10 mg/ml) were processed using a protein-folding spin-column screening kit. Lane 1, protein molecular weight markers; lanes 2–10, eluates from spin-column nos. 1–9. (B) SDS-PAGE analysis of the affinity-purified recombinant mouse α-CP2. Lane 1, protein molecular weight markers; lane 2, 5 μl of refolded and purified α-CP2 protein.

The folding conditions were extensively tested to obtain the most active α-CP2 protein. To optimize the α-CP2 protein folding conditions, we used the Spin-Column Protein Folding Screen kit (ProFoldin Protein preparation and assay technologies, Hudson, MA, USA). The ProFoldin Spin-Column was considered the most effective. We tested several refolding methods, including ProFoldin Spin-Columns, as well as others. Finally, we selected the ProFoldin Spin-Columns due to its simple folding conditions and technical simplicity. The screen kit includes nine different protein folding spin-columns (column nos. 1–9). The nine different columns represent the nine most promising folding conditions. Using the Spin-Column Protein Folding Screen kit, we identified column no. 8 as the one with the optimal protein folding conditions (Fig. 2A, lane 9). Ni-NTA His-binding resin affinity chromatography was employed to purify the insoluble recombinant α-CP2 under 8 M urea. The denatured, purified α-CP2 protein was folded using the Spin-Column Protein Folding Screen kit (column no. 8). The SDS-PAGE results revealed that the α-CP2 protein was folded and purified (Fig. 2B, lane 2).

### RNA binding property of α-CP2

**Figure 3:**
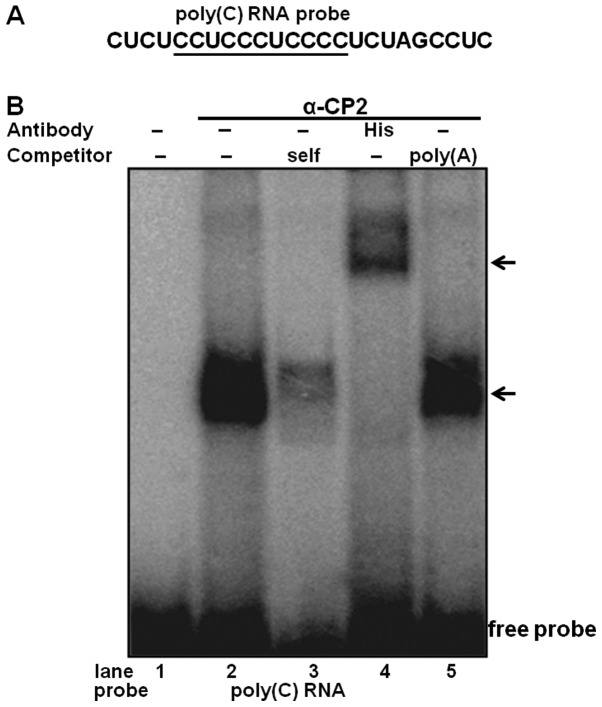
RNA electrophoretic mobility shift assay (EMSA) of recombinant mouse His-tagged α-complex protein 2 (α-CP2) using an RNA probe. (A) The sequence of RNA containing a poly(C) sequence. (B) EMSA was performed on purified recombinant α-CP2 using a [^32^P]-labeled RNA poly(C) sequence as a probe. Lane 1, probe alone; lane 2, purified α-CP2 without antibody; lane 3, self-competition; lane 4, purified α-CP2 protein with an anti-His antibody; lane 5, purified α-CP2 protein with a poly(A) competitor. The α-CP2/poly(C) and α-CP2/poly(C)/antibody complexes are indicated by arrows.

To determine the physical interaction of purified α-CP2 with the RNA poly(C) sequence, an RNA EMSA was performed using purified α-CP2 and ^32^P-labeled RNA (Fig. 3A). The purified α-CP2 protein was able to shift the target RNA probe (Fig. 3B, lane 4). The specificity of this RNA-protein interaction was verified by competitive inhibition in the presence of a 100-fold excess of an unlabeled self-competitor (Fig. 3B, lane 3) and a poly(A) sequence of the same length as the competitor (Fig. 3B, lane 5). We also used an anti-His antibody with the purified α-CP2 protein, which was His-tagged from the pET21b-α-CP2 plasmid. The formation of the α-CP2-RNA complex was abolished by the addition of the anti-His antibody and supershifted (Fig. 3B, lane 4), indicating a specific interaction between α-CP2 and the RNA poly(C) sequence.

### Single-stranded DNA binding property of purified α-CP2

**Figure 4:**
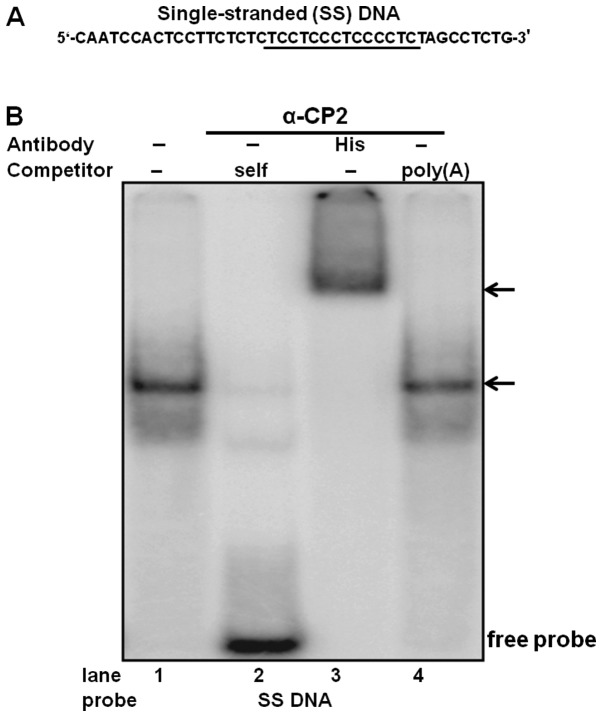
DNA electrophoretic mobility shift assay (EMSA) of recombinant mouse His-tagged α-complex protein 2 (α-CP2) using a single-stranded DNA probe. (A) The sequence of the single-stranded DNA containing a C-rich sequence. (B) EMSA was performed on purified recombinant α-CP2 using a [^32^P]-labeled single-stranded DNA poly(C) sequence as a probe. Lane 1, purified α-CP2 without antibody; lanes 2, self-competition; lane 3, purified α-CP2 protein with an anti-His antibody; lane 4, purified α-CP2 protein with a poly(A) competitor. The α-CP2/poly(C) and α-CP2/poly(C)/antibody complexes are indicated by arrows.

To determine the physical interaction of purified α-CP2 with the single-stranded DNA C-rich sequence, DNA EMSA was performed using purified α-CP2 protein and a ^32^P-labeled single-stranded DNA oligonucleotide (Fig. 4A). The specificity of this DNA-protein interaction was verified by competitive inhibition in the presence of a 100-fold excess of an unlabeled self-competitor (Fig. 4B, lane 2) and a poly(A) sequence of the same length as the competitor (Fig. 4B, lane 4). We also used an anti-His antibody with the purified α-CP2 protein, which was His-tagged from the pET21b-α-CP2 plasmid. The formation of the α-CP2-DNA complex was abolished by the addition of the anti-His antibody and supershifted (Fig. 4B, lane 3), indicating a specific interaction between α-CP2 and the single-stranded DNA C-rich sequence.

### S1 nuclease sensitivity of promoter DNA containing single-stranded poly(C) sequences

**Figure 5:**
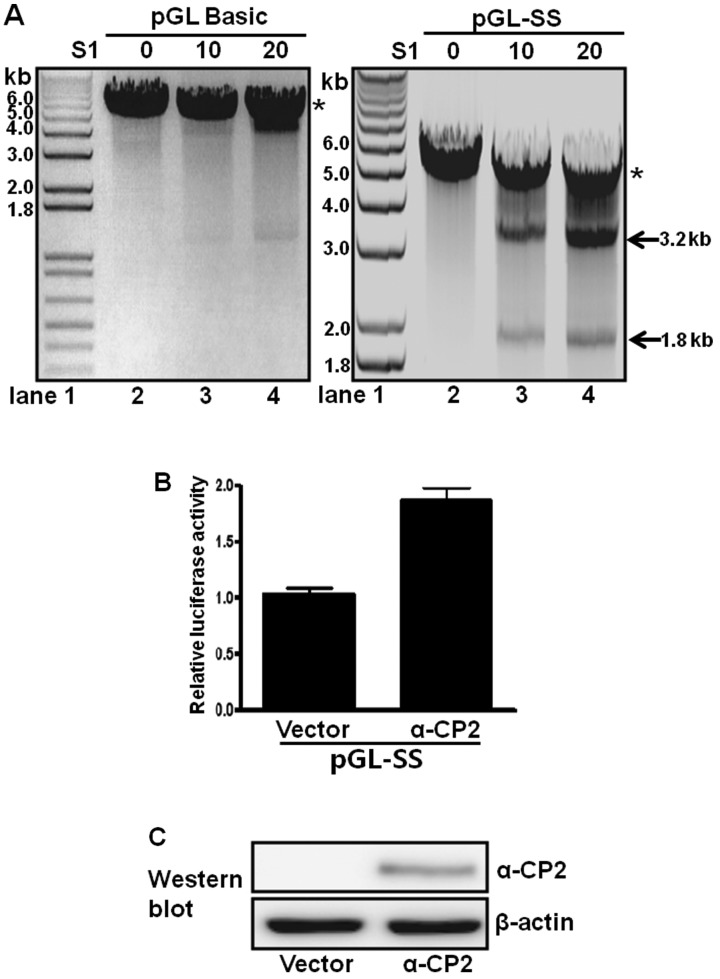
The S1 nuclease sensitivity of single-stranded poly(C)-containing promoters. (A) The single-stranded poly(C) sequence promoter fused with the promoterless pGL3-basic vector (pGL-SS) and pGL3-basic (control) were treated with vehicle (lane 2 in each panel) and increasing amounts of S1 nuclease (lanes 3–4 in each panel), followed by digestion with the *Xba*I restriction enzyme. Lane 1, molecular size markers (1 kb ladder). The *Xba*I-linearized plasmid (asterisk) and the 3.2- and 1.8-kb fragments are indicated by arrows. (B) α-complex protein 2 (α-CP2) activates the single-stranded poly(C) sequence-containing promoter (pGL-SS). The luciferase activity is expressed as n-fold relative to the activity of the luciferase reporter transfected with vector alone (assigned an activity value of 1.0). The transfection efficiencies were normalized against β-galactosidase activity. These data are representative of three independent experiments with at least two different plasmid preparations. Bars indicate the range of the standard errors. (C) The relative levels of α-CP2 protein expression following plasmid transfection were confirmed by immunoblot analysis (left lane, vector DNA alone; right lane, α-CP2 plasmid DNA). Immunoblots against β-actin were performed as an internal control.

Single-stranded regions resulting from the non-B DNA form, such as melting DNA or an intramolecular triplex structure, are accessible to single-stranded-sensitive nucleases (e.g., S1 nuclease) at low concentrations (28). Accordingly, a pGL-SS plasmid containing a poly(C) sequence inserted into the promoterless pGL3-basic plasmid has promoter activity and was examined for its S1 nuclease sensitivity. The plasmid was treated with or without S1 nuclease and then digested with *Xba*I. In the absence of S1 nuclease treatment, only a 5-kb *Xba*I-linearized DNA band was observed in the digested pGL-SS sample (Fig. 5A, lane 2 in right panel). However, treatment with both the S1 nuclease and *Xba*I produced two DNA fragments of 1.8 and 3.2 kb, and the intensity of both bands increased with increasing amounts of S1 nuclease (Fig. 5A, lanes 3 and 4, right panel). These results suggest the presence of a single-stranded poly(C) sequence located approximately 1.8 kb from the *Xba*I site (Fig. 5A, right panel). When the promoterless pGL3-basic plasmid was examined using the same digestion procedures (Fig. 5A, left panel), only the 5-kb linearized band was observed, with or without S1 nuclease treatment. These results confirmed that the poly(C) sequence of pGL-SS is present as a single-stranded DNA (Fig. 5A, right panel).

### α-CP2 transcriptionally regulates a single-stranded poly(C)-containing promoter

The role of α-CP2 binding to the poly(C)-containing promoter was examined by fusing the single-stranded promoter with a luciferase reporter and co-transfecting these constructs with an α-CP2 expression plasmid (pcDNA4Myc-HisA-α-CP2) into neuronal NS20Y cells. α-CP2 activated approximately 80% of the activity of the single-stranded promoter (Fig. 5B) compared with the cells transfected with the pcDNA4 vector alone. Immunoblot analyses with anti-Myc were performed following plasmid transfection to confirm the overexpression of the α-CP2 protein. β-actin was used as an internal control. The protein levels of α-CP2 were increased, whereas the protein levels of α-CP2 were not detectable in the cells transfected with the pcDNA4 vector alone (negative control) (Fig. 5C). These results indicate that α-CP2 acts as a transcriptional activator of promoters containing single-stranded poly(C) sequences.

## Discussion

α-CP2 belongs to a family of KH domain-containing proteins that specifically interact with poly(C) DNA/RNA sequences and require three C-rich motifs (underlined) with a few intervening nucleotides (29), such as α-globin mRNA, 5′-CCCAACGGGCCCU CCUCCC-3′; folate receptor mRNA, 5′-CUCCAUUCCC ACUCCCU-3′; 15-LOX mRNA CCCCACCCUCUUCCCC AAG-3′ (30–32). α-CP1 (PCBP1) has been shown to bind specifically to the single-stranded DNA element of the proximal promoter region in the mouse mu opioid receptor gene and activate the gene (28). As previously demonstrated, hnRNP K binds specifically to the double-stranded DNA element of TC1 and TC2 and induces a single-stranded conformation, in addition to binding to the single-stranded TC3 sequence of the SRC1A gene promoter (33). Moreover, in a previous study, we demonstrated that hnRNP K and α-CP3 (PCBP3) can specifically bind to single-stranded and double-stranded DNA elements and may thus act as a transcriptional regulator (34).

A previous study demonstrated that the soluble form of α-CP2 can be produced in *E. coli* JM109 cells grown at 30°C overnight with IPTG (35). However, the same culture and induction conditions produced inclusion bodies of the α-CP2 protein. To overcome this problem, α-CP2 was affinity-purified under denatured conditions and refolded using the protein-folding spin-columns (34). To our knowledge, this is the first study to demonstrate the purification, solubilization and folding of α-CP2, and the production of a functionally active form for RNA/DNA EMSA analysis using the *E. coli* protein expression system.

In this study, we investigated the RNA/DNA binding properties of α-CP2. α-CP2, a member of the PCBP family, binds to the RNA/DNA poly(C) elements. Specific interactions between α-CP2 and RNA/DNA poly(C) sequences were observed using RNA/DNA EMSA assays. The EMSA results demonstrated a sequence-specific interaction between solubilized α-CP2 and the poly(C) rich RNA/DNA sequences. The poly(C) rich sequence is enough for the adaption of a single-stranded DNA conformation of the promoter region in the mouse mu opioid receptor gene (28). The single-stranded DNA conformation was sensitive to S1 nuclease digestion. An equilibrium between the double-stranded DNA and the single-stranded DNA (melted DNA structure) may exist, such that a small portion of the single-stranded DNA structure can be digested by S1 nuclease (28). When the single-stranded DNA construct (pGL-SS) containing a poly(C) sequence and a plasmid expressing α-CP2 were co-transfected, α-CP2 activated the expression of a luciferase reporter. To our knowledge, this study demonstrates for the first time that α-CP2 acts as a transcriptional activator by binding to a single-stranded poly(C) sequence.

## References

[1] Du Z, Lee JK, Fenn S, Tjhen R, Stroud RM, James TL (2007). X-ray crystallographic and NMR studies of protein-protein and protein-nucleic acid interactions involving the KH domains from human poly(C)-binding protein-2. RNA.

[2] Makeyev AV, Liebhaber SA (2002). The poly(C)-binding proteins: a multiplicity of functions and a search for mechanisms. RNA.

[3] Leffers H, Dejgaard K, Celis JE (1995). Characterisation of two major cellular poly(rC)-binding human proteins, each containing three K-homologous (KH) domains. Eur J Biochem.

[4] Kiledjian M, Wang X, Liebhaber SA (1995). Identification of two KH domain proteins in the alpha-globin mRNP stability complex. Embo J.

[5] Makeyev AV, Liebhaber SA (2000). Identification of two novel mammalian genes establishes a subfamily of KH-domain RNA-binding proteins. Genomics.

[6] Makeyev AV, Eastmond DL, Liebhaber SA (2002). Targeting a KH-domain protein with RNA decoys. RNA.

[7] Du Z, Lee JK, Tjhen R (2005). Crystal structure of the first KH domain of human poly(C)-binding protein-2 in complex with a C-rich strand of human telomeric DNA at 1.7 A. J Biol Chem.

[8] Sidiqi M, Wilce JA, Vivian JP (2005). Structure and RNA binding of the third KH domain of poly(C)-binding protein 1. Nucleic Acids Res.

[9] Chkheidze AN, Liebhaber SA (2003). A novel set of nuclear localization signals determine distributions of the alphaCP RNA-binding proteins. Mol Cell Biol.

[10] Meng Q, Rayala SK, Gururaj AE, Talukder AH, O'Malley BW, Kumar R (2007). Signaling-dependent and coordinated regulation of transcription, splicing, and translation resides in a single coregulator, PCBP1. Proc Natl Acad Sci USA.

[11] Shi H, Bencze KZ, Stemmler TL, Philpott CC (2008). A cytosolic iron chaperone that delivers iron to ferritin. Science.

[12] Funke B, Zuleger B, Benavente R (1996). The mouse poly(C)-binding protein exists in multiple isoforms and interacts with several RNA-binding proteins. Nucleic Acids Res.

[13] Blyn LB, Swiderek KM, Richards O, Stahl DC, Semler BL, Ehrenfeld E (1996). Poly(rC) binding protein 2 binds to stem-loop IV of the poliovirus RNA 5′ noncoding region: identification by automated liquid chromatography-tandem mass spectrometry. Proc Natl Acad Sci USA.

[14] You F, Sun H, Zhou X (2009). PCBP2 mediates degradation of the adaptor MAVS via the HECT ubiquitin ligase AIP4. Nat Immunol.

[15] Zhu J, Chen X (2000). MCG10, a novel p53 target gene that encodes a KH domain RNA-binding protein, is capable of inducing apoptosis and cell cycle arrest in G(2)-M. Mol Cell Biol.

[16] Castaño Z, Vergara-Irigaray N, Pajares MJ, Montuenga LM, Pio R (2008). Expression of alpha CP-4 inhibits cell cycle progression and suppresses tumorigenicity of lung cancer cells. Int J Cancer.

[17] Pio R, Zudaire I, Pino I (2004). Alpha CP-4, encoded by a putative tumor suppressor gene at 3p21, but not its alternative splice variant alpha CP-4a, is underexpressed in lung cancer. Cancer Res.

[18] Weiss IM, Liebhaber SA (1995). Erythroid cell-specific mRNA stability elements in the alpha 2-globin 3′ nontranslated region. Mol Cell Biol.

[19] Blyn LB, Towner JS, Semler BL, Ehrenfeld E (1997). Requirement of poly(rC) binding protein 2 for translation of poliovirus RNA. J Virol.

[20] Gamarnik AV, Andino R (1997). Two functional complexes formed by KH domain containing proteins with the 5′ noncoding region of poliovirus RNA. RNA.

[21] Collier B, Goobar-Larsson L, Sokolowski M, Schwartz S (1998). Translational inhibition in vitro of human papillomavirus type 16 L2 mRNA mediated through interaction with heterogenous ribonucleoprotein K and poly(rC)-binding proteins 1 and 2. J Biol Chem.

[22] Ostareck DH, Ostareck-Lederer A, Shatsky IN, Hentze MW (2001). Lipoxygenase mRNA silencing in erythroid differentiation: The 3′UTR regulatory complex controls 60S ribosomal subunit joining. Cell.

[23] Andino R, Boddeker N, Silvera D, Gamarnik AV (1999). Intracellular determinants of picornavirus replication. Trends Microbiol.

[24] Patterson SD, Aebersold R (1995). Mass spectrometric approaches for the identification of gel-separated proteins. Electrophoresis.

[25] Kim CS, Hwang CK, Song KY (2008). Novel function of neuron-restrictive silencer factor (NRSF) for posttranscriptional regulation. Biochim Biophys Acta.

[26] Hwang CK, Wu X, Wang G, Kim CS, Loh HH (2003). Mouse mu opioid receptor distal promoter transcriptional regulation by SOX proteins. J Biol Chem.

[27] Choi HS, Hwang CK, Kim CS (2005). Transcriptional regulation of mouse mu opioid receptor gene: Sp3 isoforms (M1, M2) function as repressors in neuronal cells to regulate the mu opioid receptor gene. Mol Pharmacol.

[28] Ko JL, Loh HH (2001). Single-stranded DNA-binding complex involved in transcriptional regulation of mouse mu-opioid receptor gene. J Biol Chem.

[29] Du Z, Fenn S, Tjhen R, James TL (2008). Structure of a construct of a human poly(C)-binding protein containing the first and second KH domains reveals insights into its regulatory mechanisms. J Biol Chem.

[30] Waggoner SA, Liebhaber SA (2003). Regulation of alpha-globin mRNA stability. Exp Biol Med (Maywood).

[31] Ostareck-Lederer A, Ostareck DH, Standart N, Thiele BJ (1994). Translation of 15-lipoxygenase mRNA is inhibited by a protein that binds to a repeated sequence in the 3′ untranslated region. EMBO J.

[32] Xiao X, Tang YS, Mackins JY (2001). Isolation and characterization of a folate receptor mRNA-binding trans-factor from human placenta. Evidence favoring identity with heterogeneous nuclear ribonucleoprotein E1. J Biol Chem.

[33] Ritchie SA, Pasha MK, Batten DJ (2003). Identification of the SRC pyrimidine-binding protein (SPy) as hnRNP K: implications in the regulation of SRC1A transcription. Nucleic Acids Res.

[34] Kang DH, Song KY, Choi HS, Law PY, Wei LN, Loh HH (2012). Novel dual-binding function of a poly (C)-binding protein 3, transcriptional factor which binds the double-strand and single-stranded DNA sequence. Gene.

[35] Bedard KM, Walter BL, Semler BL (2004). Multimerization of poly(rC) binding protein 2 is required for translation initiation mediated by a viral IRES. RNA.

